# The Content of Conjugated Linoleic Acid and Vaccenic Acid in The Breast Milk of Women from Gdansk and The Surrounding District, As Well As In, Infant Formulas and Follow-up Formulas. Nutritional Recommendation for Nursing Women

**DOI:** 10.34763/devperiodmed.20182202.128134

**Published:** 2018-06-30

**Authors:** Dorota Martysiak-Żurowska, Bogumiła Kiełbratowska, Agnieszka Szlagatys-Sidorkiewicz

**Affiliations:** 1 Department of Chemistry, Technology and Biotechnology of Food; Faculty of Chemistry, Gdansk University of Technology, Gdansk, Poland; 2 Department of Obstetrics; Medical University of Gdansk, Gdansk Poland; 3 Department of Pediatrics, Gastroenterology, Hepatology and Nutrition, Medical University of Gdansk, Gdansk Poland

**Keywords:** breast milk, conjugated linoleic acid, vaccenic acid, diet, supplementation, mleko kobiece, skoniugowany kwas linolowy, kwas wakcenowy, dieta, suplementacja

## Abstract

Fatty acids are some of the most important components of human milk. Polyunsaturated fatty acids (PUFAs) are essential nutrients required for the optimal growth and development of infants, especially the central nervous system, brain and retina.

**Aim:**

To determine the conjugated linolenic acid (CLA) and vaccenic acid (VA) content of human breast milk from mothers consuming different diets, and to compare the results with CLA and VA levels in infant formulas (IF) and follow-up formulas (FF).

**Material and methods:**

Fifty healthy mothers were classified according to their diet status into one of two groups: diet low in dairy products and conventional diet without limiting the intake of dairy products. Dietary intake of dairy fat was determined based on 3-day food diaries. Fatty acid (FA) composition in samples were analyzed by High Resolution Gas Chromatography (HR-GC).

**Results:**

In the group of 20 mothers whose diets were deficient in dairy products, the average CLA content of breast milk fat was determined to be 0.27% of total FA, the VA 0.36%. In the group of 30 women consuming dairy products, the average content of CLA and VA in breast milk fat was statistically significantly higher: 0.49% and 0.69% of total FAs, respectively. In the fat of the IF and FF tested (n=11) only trace amounts of both FA were found.

**Conclusion:**

The results of the study indicate that CLA and VA concentrations of human milk can be influenced by diet. It is recommended that the source of these FAs in the diet of breastfeeding women are natural products and not dietary supplements. The majority of commercially available IF and FF do not contain sufficient amounts of CLA and VA, and that their FA composition is deficient in comparison with breast milk fat.

## Streszczenie

Kwasy tłuszczowe są jednym z najważniejszych składników mleka ludzkiego. Wielonienasycone kwasy tłuszczowe PUFA są składnikami odżywczymi niezbędnymi do optymalnego wzrostu i rozwoju niemowląt, zwłaszcza ośrodkowego układu nerwowego, mózgu i siatkówki oka.

**Celem pracy:**

było określenie zawartości izomerów skoniugowanego kwasu linolowego (CLA) i kwasu wakcenowego (VA) w mleku kobiet, których diety znacząco różniły się spożyciem tłuszczu mlecznego oraz porównanie wyników z poziomem CLA i VA w popularnych mieszankach mlekozastępczych do początkowego i dalszego żywienia niemowląt.

**Materiał i metody:**

Do badań zakwalifikowano pięćdziesiąt zdrowych matek, które sklasyfikowano zgodnie z ich dietą do jednej z dwóch grup: dieta uboga w produkty mleczne i dieta bez ograniczenia spożycia mleka i jego przetworów. Dzienne spożycie tłuszczu mlecznego przez kobiety określono na podstawie 3-dniowego wywiadu żywieniowego. Zawartość kwasów tłuszczowych (KT) w badanych próbkach oznaczono techniką wysokorozdzielczej chromatografii gazowej (HR-GC).

**Wyniki:**

W grupie 20 kobiet spożywających dietę ubogą w produkty mleczne zawartość CLA w tłuszczu mleka wynosiła średnio 0,27% ogólnego składu KT, VA średnio 0,36%. W grupie 30 kobiet spożywających produkty mleczne zawartość CLA i VA w tłuszczu mleka była statystycznie istotnie wyższa i wynosiła średnio: CLA 0,49%, VA 0,69%. W tłuszczu badanych mieszanek modyfikowanych (n=11) stwierdzono zaledwie śladowe ilości obu KT.

**Wnioski:**

Wyniki badania wskazują, że dieta może wpływać na stężenie CLA i VA w tłuszczu mleka ludzkiego. Zaleca się, aby źródłem tych FA w diecie kobiet karmiących były produkty naturalne, a nie suplementy diety. Najpopularniejsze mieszanki modyfikowane dostępne w obrocie detalicznym nie zawierają w swym składzie odpowiednich ilości CLA i VA, a tym samym nie oddają w pełni składu KT mleka ludzkiego.

## Background

The positional and geometrical *cis* and *trans* isomers of octadecadienoic acid (C18:2) with conjugated double bonds are collectively referred to as conjugated linoleic acids (CLAs). CLAs are the indirect hydrogenation product of polyunsaturated fatty acids (PUFA) – linoleic acid (LA, C18:2 9c, 12c) and alpha-linolenic acid (ALA, C18:3 9, 12, 15 all *cis*) – in the digestive tract of ruminants. The biohydrogenation process begins with LA isomerization by the enzymes produced by *Butyrivibrio fibrisolvens*. The produced molecule of conjugated diene of LA is converted to vaccenic acid (VA, C18:1 11*-trans*) and elaidic acid (EA, C18:1 9*-trans*). However, not all CLA molecules are converted to monoenoic FAs. Some CLAs are absorbed and transported to milk fat, intramuscular fat and adipose tissue [1]. The most prevalent CLA in ruminant meat and milk is rumenic acid (RA), a 9-cis 11-trans isomer of CLA. RA represents from 73% to 93% of total CLAs present in fat from the tissue and milk of ruminants [1, 2]. The best sources of CLA is beef meat (1.2-10.0 mg CLA/g fat), lamb meat (4.3-11.0 mg CLA/g fat) and cow’s milk fat which is characterized by the highest content of CLA, from 2 to 37 mg/g [3].

This group of fatty acids (FA) has attracted considerable interest when research studies demonstrated that CLA is one of the few chemical compounds of animal origin whose biological activity delivers health benefits. CLA has strong anticarcinogenic properties against various types of cancer in the different stages of disease progression, including breast cancer [2, 4, 5, 6, 7, 8]. Rumenic acid (RA) is the most potent anticarcinogenic compound [7, 9]. In experimental animals, CLA reduced atherosclerosis and total cholesterol levels by contributing to a healthy LDL/HDL ratio [10]. Previous studies of animals demonstrated that CLA can normalize glucose metabolism. However, the results of a recent meta-analysis evaluating the combined influence of 9c, 11t CLA and 12t, 10c CLA isomers on fasting blood sugar levels did not confirm these effects in humans [11]. CLA stimulates metabolism, increases muscle mass, reduces fat tissue and influences fat metabolism [12, 13, 14].

In the human body, CLA is derived mainly from dietary sources. 9*-cis*, 11-*trans* and 9*-trans*, 11-*trans* CLA isomers were found in human adipose tissue, which also contained trace amounts of 9*-trans*, 11-*cis* and 9*-cis*, 11-*cis* isomers [15]. Dietary supplementation with LA does not increase CLA levels in the blood plasma, which indicates that gut bacteria are unable to isomerize LA to CLA [16]. However, VA from dietary sources increases CLA levels in the blood serum, which implies that VA is desaturated to RA in the human body [17, 18]. Δ9-desaturase transforms stearic acid C18:0 and uses VA as a substrate for enzymatic conversion, probably due to similarities in the linear structure of VA molecules. Therefore, a diet that is abundant in CLA as well as VA is a good source of conjugated octadecadienoic acid [18]. CLA isomers are also present in human breast milk, from 0.3 to 1.2 g/100g fatty acids [19].

In view of the potential health benefits of CLA, the aim of this study was to determine the CLA and VA content of human breast milk from mothers consuming different diets: i.e. a conventional diet, without limiting the intake of dairy products and a diet low in dairy products, and to compare the results with CLA and VA levels in infant formulas and follow-up formulas for children that for various reasons are not fed breast milk.

## Materials and methods

### Ethics

All of our experimental procedures have been approved by the Local Ethics Committee of the Medical University of Gdańsk. All the patients have given their written consent to participate in the study.

### Human milk

Mature human milk from mothers who consumed a milk-free diet, mainly due to their children’s allergy to bovine milk (N=20). The mothers consumed only small quantities of milk fat in the form of margarine and butter mixes or margarine and yoghurt mixes, mainly for spreading on bread. Their daily intake of dairy fat was below 4.2±0.5g.

Mature human milk from women eating a conventional diet without limiting the intake of dairy products (N=30). Their daily intake of dairy fat was higher than 12.7±0.7g.

Mature human milk was collected from mothers, residents of Gdansk, who gave birth to full-term babies without complications. The exclusion criteria included acute and chronic disorders, pharmacotherapy other than vitamin supplementation and maternal smoking. All the newborns were in good health (Apgar score ≥8 in the first minute of life), and their body weights were within the norm (3100-3800 g). Every morning milk was pumped by the mothers at home with an electric breast pump (Symphony, Medela) upon the observance of general hygiene standards. Samples of around 20 ml were collected from the milk pumped from one breast, and they were freezer stored at a temperature of around -18°C until analysis. The remaining milk was fed to the children.

### Infant (IF) and follow-up (FF) formulas

Eleven most popular infant and follow-up formulas from 4 manufacturers (labeled A, B, C and D) were analyzed. FA composition was determined in 6 infant formulas for children younger than 6 months (No. 1) and 5 follow-up formulas for children older than 6 months (No. 2), including a soy milk formula and a hypoallergenic (HA) formula containing hydrolyzed cow’s milk proteins.

### Dietary intake of dairy fat

The dietary intake of nutrients was determined directly before milk sampling based on 3-day nutritional diaries kept by the mothers. Each participant obtained detailed instructions about how to weigh and record all the foods consumed. The results were processed using Dieta 4.0 software (National Institute of Food and Nutrition, Poland) which includes a listing of 1800 dishes and foods. Milk fat intake was estimated by summing up the amounts of total fat from all the dairy products. Total energy, nutrient and fatty acid intakes for each mother were calculated as the mean daily intake.

### Analytical procedure

Lipids were extracted from breast milk (within 24 hours after collection) and commercial formulas (immediately after dissolution) by means of the Roese-Gottlieb method [20]. Fatty acid methyl ester (FAME) profiles were determined according to Standard EN:ISO 5509:2000 [21]. FAME were separated by high-performance gas chromatography (HP-GC) based on the length of the hydrocarbon chain and the degree of FAME unsaturation. Analyses were carried out using a Hewlett-Packard GC system with a flame ionization detector (FID) and the Rtx 2330 column (Restek, Bellefonte, Pennsylvania, USA). Qualitative and quantitative analyses of CLA in the samples evaluated were performed with a CLA methyl ester standard (Sigma-Aldrich). The results were expressed as the percentage of FA in the total FAs tested.

### Statistical analysis

Variance model analysis was used to assess the differences in the intake of total energy, selected nutrients, content of CLA isomers and VA in milk fat between the two diets. When the normality of the distribution of data was not met, the Kruskal-Wallis One Way Analysis of Variance on Ranks was used. (Statistica 12 Software, Poland). The results are presented as mean values and standard deviations (mean ± SD). Differences between means were considered statistically significant at the *p* < 0.05 level. Pearson’s correlation was used to analyze the relations between the content of CLA 9c,11t vs. the content of pentadecanoic acid PA (C15:0) and vaccenic acid VA (C18:1 11t) in human milk fat.

## Results

Mean intakes of total energy, protein, carbohydrate, total fat, saturated, monounsaturated and polyunsaturated fatty acids were not significantly different between the two diets (Table I). Only the consumption of dairy fat was greater in the group of women eating a standard diet, without limiting dairy product intake. The average daily milk fat intake estimated in this study group was < 12.7 g.

**Table I:** Daily intake of energy, selected nutrients, fatty acids and dairy fat (Mean ± SD) by nursing women. Tabela I. Dzienne pobranie energii ogółem, wybranych składników odżywczych, kwasów tłuszczowych oraz tłuszczu mlecznego (średnia ± SD) przez kobiety karmiące.

Daily intake *Dzienne pobranie*	Diet without limiting intake of dairy products N= 30 *Dieta bez ograniczenia spożycia mleka i jego przetworów, N=30*	Diet low in dairy products, N= 20 *Dieta uboga w mleko i jego przetwory, N= 20*
Total energy [kcal/day] *Wartość* *energetyczna*	2063.3 ± 562.61	1868.8 ± 502.45
Protein [g/day] *Białka*	81.4 ± 26.84	76.0 ± 24.72
Carbohydrate [g/day] ** Węglowodany	285.3 ± 71.42	271.8 ± 84.35
Total fat [g/day] *Tłuszcz* *ogółem*	70.0 ± 27.18	55.8± 20.99
SFA [g/day]	27.9 ± 13.52	21.8 ± 9.98
MUFA [g/day]	26.1 ± 11.40	21.8 ± 8.23
PUFA [g/day]	8.9 ± 3.76	7.2 ± 3.30
Dairy fat [g/day] *Tłuszcz mleczny*	>12.7 ± 0.7^a^	<4.2 ± 0.5^a^

a Statistically significant values differing between the diets (*p*<0.05) Wartości różniące się istotne statystycznie pomiędzy dietami (p<0.05). SFA – Saturated Fatty Acid; MUFA Monounsaturated Fatty Acids; PUFA – Polyunsaturated Fatty Acids SFA – nasycone kwasy tłuszczowe; MUFA jednonienasycowne kwasy tłuszczowe; PUFA – wielonienasycone kwasy tłuszczowe

All the samples of fat from mature breast milk contained CLA isomers. In the group of mothers who consumed a standard diet, the CLA content of milk fat ranged from 0.35% to 0.64% of total FAs (0.49 ± 0.08% on average). In the group of women who limited their consumption of dairy products on account of their children’s allergy to cow’s milk, the CLA content of milk fat ranged from 0.14% to 0.41% (0.27% on average) (Table II). In both groups, RA was the predominant CLA which accounted for 72-99% of all CLA isomers in the milk fat samples examined. The second most abundant CLA was isomer 10t, 12c which accounted for 10-19% of total CLAs.

**Table II:** The content of CLA isomers and VA [% total fatty acids] in breast milk fat from mothers consuming dairy products and mothers whose diets were deficient in these products. Tabela II. Zawartość izomerów CLA i VA [% ogólnej zawartości KT] w tłuszczu mleka matek spożywających bez ograniczeń mleko i jego przetwory oraz matek pozostających na diecie ubogiej w te produkty.

Fatty acid *Kwas tłuszczowy*	Diet without limiting intake of dairy products N= 30 *Dieta bez ograniczenia spożycia mleka i jego przetworów, N=30*		Dairy fat deficient diet, N= 20 *Dieta uboga w mleko i jego przetwory, N= 20*	
	mean ±SD *średnia ±SD*	range *zakres*	mean ±SD *średnia ±SD*	range *zakres*
CLA c9,t11	0.40^a^±0.084	0.32-0.57	0.22^a^±0.062	0.14-0.33
CLA t10,c12	0.05±0.012	0.03-0.07	0.04±0.017	ND-0.07
other CLAs inne CLA	0.03±0.019	ND-0.07	0.03±0.004	ND-0.04
Total CLAs Suma CLA	0.49^a^±0.079	0.35-0.64	0.27^a^±0.071	0.14-0.41
VA	0.69^a^±0.219	0.34-1.03	0.36^a^±0.100	0.20-0.48

* ND – non-detectable ND – poniżej poziomu wykrywalności CLA – conjugated linolenic acid, VA – vaccenic acid CLA – skoniugowany kwas linolowy, VA – kwas wakcenowy ^a^ Values between the diets differed in a statistically significant way (*p*<0.05) Wartości różniące się pomiędzy dietami istotne statystycznie (p<0.05).

The remaining CLA isomers (9t,11c; 9t,11t; 10c,12c) represented 6-8% of the CLAs identified in milk samples. The content of RA (MD 0.18; 95% CI: 0.1359– 0.2241, p<0.001) and total CLA isomers (MD 0.22; 95% CI: 0.1759-0.2641, p<0.001) was statistically higher in the milk of women eating a standard diet than in the milk of women consuming a diet deficient in cow’s milk fat.

The content of VA in breast milk fat was also highly correlated with the mothers’ intake of products rich in cow’s milk fat. The concentration of VA was significantly higher in the milk of women with a standard diet than in the milk of mothers who limited their intake of dairy products (0.69% vs. 0.36% of total FAs respectively, p<0,001).

The relatively strong positive correlations between the RA content of human milk fat and the content of other FAs typically found in milk fat, i.e. VA (r=0.662, p<0.05) and pentadecanoic acid PA C15:0 (r=0.754, p<0.05), provide evidence that CLA isomers in breast milk originate from diets rich in dairy products (Figure I). Similarly to CLA and VA, PA is synthesized by the ruminal microbiota in cattle. PA is not synthesized in the human body, and it is not effectively metabolized, because a PA molecule has an odd number of carbon atoms [22].

**Fig. 1:**
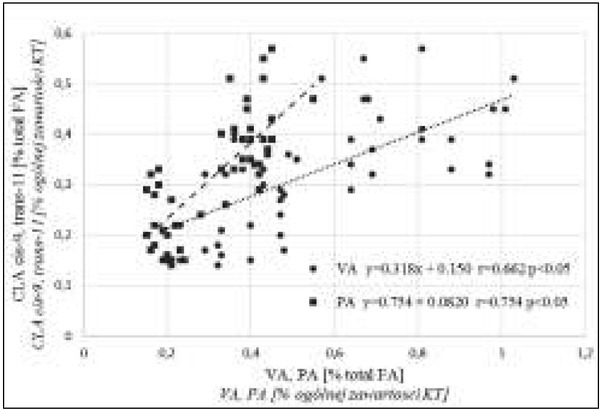
Correlations between the content of rumenic acid RA (CLA 9c,11t) vs. the content of pentadecanoic acid PA (C15:0) and vaccenic acid VA (C18:1 11t) in human milk fat. Ryc. 1. Zależność pomiędzy zawartością kwasu żwaczowego RA (CLA 9c, 11t) a zawartością kwasu pentadekanowego PA (C15:0) oraz kwasu wakcenowego VA (C18:1 11t) w tłuszczu mleka ludzkiego.

Only trace amounts of two CLA isomers: RA and 10t,12c isomer, were detected in the infant formulas evaluated. RA accounted for up to 0.04% of total FAs. The analyzed formulas also contained VA (up to 0.05% of total FAs) (Table III). In 3 out of the 11 tested products, the content of CLA isomers was below the detection limit (0.01% of total FAs). The presence of CLA in IF and FF can be attributed to the fact that the formulas examined contain skimmed cow’s milk which may contain trace amounts of the analyzed FAs.

**Table III:** The content of CLA isomers and VA in the analyzed infant formulas IF (1) and follow-up formulas FF (2) [% total FAs]. Table III. Zawartość izomerów CLA i VA w tłuszczu analizowanych mieszanek do początkowego (1) i dalszego (2) żywienia niemowląt [% ogólnego składu KT].

Infant formulas *Mieszanka mlekozastępcza*	CLA 9c,11t	CLA 10t,12c	Total CLAs *Suma CLA*	VA
	mean średnia		mean ± SD *średnia ± SD*	
A*.1.	0.03	0.01	0.04 ± 0.003	0.06 ± 0.004
A.2.	0.04	ND	0.04 ± 0.003	0.05 ± 0.003
B.1.	0.02	0.02	0.04 ± 0.003	0.05 ± 0.003
B.2.	0.03	0.02	0.05 ± 0.004	0.03 ± 0.002
B.1. soy based *sojowa*	ND	ND	ND**	0.01 ± 0.001
C.1.	0.03	ND	0.03 ± 0.001	0.04 ± 0.004
C.2.	0.02	ND	0.02 ± 0.002	0.05 ± 0.002
C.1. HA*	0.01	ND	0.02 ± 0.002	0.03 ± 0.002
C.2. HA	0.01	ND	0.02 ± 0.002	0.03 ± 0.002
D.1.	ND	ND	ND	0.03 ± 0.002
D.2.	ND	ND	ND	0.03 ± 0.002

* A, B, C, D – different manufacturers * A, B, C, D – różni producenci HA – hypoallergenic HA – hipoalergiczne **ND – non-detectable **ND – poniżej poziomu wykrywalności

## Discussion

The results of this study confirm that the CLA content of human breast milk is highly correlated with the mother’s diet. In the group of women whose diets were deficient in cow’s milk and dairy products, the CLA content of breast milk accounted for less than 55% of the CLA content determined in the breast milk samples collected from women consuming dairy products.

CLA concentration in cow’s milk ranges from 2.3 to 7.2 mg/g of fat, depending on the cattle’s diet [23]. High-fat dairy products are more abundant in CLA whose content reaches 8.1-9.5 mg/g of fat in butter and 12.4-14.0 mg/g of fat in ripened cheese [3]. In the groups of respondents evaluated, the consumption of CLA isomers was determined during nutritional interviews and based on average CLA levels in various food products. Daily CLA intake was estimated at 38 mg, but not more than 60 mg, in the group of women consuming a diet deficient in dairy products, and at 115 mg, but not less than 92 mg, in the group of women eating a standard diet. According to research, breast milk is enriched with CLA around 28 hours after the consumption of foods abundant in these fatty acids [19].

For millennia, human infants have been fed mother’s milk or the milk of domesticated ruminants. For this reason, CLAs, mainly RA, as well as other milk fat components, such as cholesterol, VA and sphingomyelins, are natural ingredients in infant diets. Unfortunately, cow’s milk proteins have allergizing properties. After diagnosing a child’s allergy to cow’s milk protein, many women simply stop breast-feeding and start using a soy protein-based infant formula. Many of them just do not know that they can limit the presence of an allergy factor in breast milk by changing their diet. Women who do not consume dairy products should supplement their diets with CLA from other sources, such as butter, ruminant meat and the milk of other mammals, including goat and sheep milk, which do not cause allergic reactions. However, the milk of other ruminants contains antigens similar to those found in cow’s milk, therefore, cross-reactivity with proteins from other types of milk may occur over time.

Natural products should be the main source of CLA in the diet of nursing women, in particular products where healthy RA accounts for 73-98% of total CLAs. Another CLA isomer, fatty acid C18:2 10t,12c, which is found in substantial amounts in CLA dietary supplements, decreases the fat content of human breast milk [24,25]. There is evidence to indicate that CLA isomer 10t,12c can increase the risk of lipodystrophy and insulin resistance in humans [25]. For this reason, CLA supplements are not recommended, in particular for nursing women, until the health implications of this CLA isomer have been comprehensively researched [26].

In ruminant milk, CLA 10t,12c accounts for less than 10% of all CLA isomers. In this study, the daily consumption of CLA 10t,12c did not exceed 6 mg in women with a milk-free diet, and it was estimated at 11 mg in the group of women consuming a standard diet. CLA supplements are much more abundant in the above isomer whose content can exceed 500 mg in a daily dose. The intake of dairy fat would have to be increased to at least 700 g to supply an equivalent dose of CLA 10t,12c.

VA is also an important component of human breast milk. According to Adlof et al. [17] and Turpeinen et al. [18], VA, a *trans* FA that occurs naturally in cow’s milk, can be converted to RA in the human body. Naturally occurring *trans* FAs (VA) can be converted to CLA in the human body, which clearly differentiates them from synthetic *trans* FAs. The latter are found in hydrogenated fats and are harmful for health. The development of production technology has contributed to a significant reduction in *trans* FA levels in non-dairy spreads. However, hydrogenated fats, such as bakery shortening, still contain substantial amounts of *trans* FAs. Hidden fats in foods, including confectionary products, bakery shortening, snacks, pastry and fast foods, are the main source of synthetic *trans* FAs in the human diet [27].

Infant formulas have been used in human nutrition for a relatively short time (3 to 4 generations). The composition of IFs should resemble breast milk as closely as possible, and all the differences should be minimized. Despite the above, recent industrial practices seem to deliver the opposite effect. Milk fat in infant formulas is being completely replaced with vegetable oil. In this study, cow’s milk fat was not found in any of the analyzed IF and FF, which therefore contained only trace amounts of CLA. Vegetable oils, including palm, rapeseed, coconut, sunflower and single cell (algal) oils, are the main sources of fat in IF and FF. Nutritional guidelines relating to daily CLA intake have not been developed to date, and optimal levels of CLA isomers in the human diet have not been determined. According to research, natural food products should be the main source of CLAs [3, 19, 24, 25].

Further research is needed to investigate the role of CLAs in the human diet. The results of studies analyzing the biological activity of RA are highly promising, therefore the elimination of CLA isomers from infant formulas should be discouraged, because the resulting products do not cater to the nutritional needs of growing infants. The aim of recent industrial practices is to simplify the production process rather than develop products with nutritional benefits. Infant formulas are the only source of food for children allergic to milk. Formulas deprived of milk fat do not contain CLAs or VA, which is a substrate for the synthesis of CLA isomers.

## Summary

The RA content of human breast milk can be increased by consuming foods that are abundant in these isomers. Natural products that are rich in FAs, including butter, ripened cheese and the meat of ruminants, are the recommended sources of CLA in the diets of nursing mothers. High-fat dairy products and ruminant meat are rich sources of CLAs, in particular RA, for children who are strongly allergic to cow’s milk proteins. Dietary supplements are not recommended due to high levels of C18:2 10t,12c, one of the CLA isomers, whose health benefits have not been confirmed. CLA supplements contain significantly higher levels of 10t,12c CLA isomers than natural foods.

Unlike breast milk fat, the fat isolated from commercially available IFs does not contain CLAs. The results of the study indicate that the majority of commercially available IF and FF do not contain sufficient amounts of RA and VA, and that their FA composition is deficient in comparison with breast milk. The replacement of cow’s milk fat with coconut oil or palm oil in IF and FF should be discouraged, because it does not cater to the nutritional needs of growing infants.

## References

[1] Bauman DE, Baumgard LH, Corl BA, Griinari JM (2000). Biosynthesis of conjugated linoleic acid in ruminants. J Anim Sci.

[2] Jahreis G, Fritsche J, Mockel P, Schone F, Moller U, Steinhart H (1999). The potential anticarcinogenic conjugated linoleic acid cis-9, trans-11 C18:2, in milk of different species: cow, goat, ewe, sow, mare, woman. Nutr Res.

[3] Kowalska M, Cichosz G (2013). Dairy products – the best sources of CLA. (In Polish). Bromat Chem i Toksykol.

[4] Parodi PW (1997). Cows’ milk fat components as potential anticarcinogenic agents. J Nutr.

[5] Shultz TD, Chew BP, Seaman WR (1992). Differential stimulatory and inhibitory responses of human MCF-7 breast cancer cells to linoleic acid and conjugated linoleic acid in culture. Anticancer Res.

[6] Ip C, Scimeca JA, Thompson HJ (1994). Conjugated linoleic acid. A powerful anticarcinogen from animal fat sources. Cancer.

[7] Kelley NS, Hubbard NE, Erickson KL (2007). Conjugated linoleic acid isomers and cancer. J Nutr.

[8] Lee KW, Lee HJ, Cho HY, Kim YJ (2005). Role of the conjugated linoleic acid in the prevention of cancer. Crit Rev Food Sci Nutr.

[9] Kramer JK, Parodi PW, Jensen RG, Mossoba MM, Yurawecz MP, Adlof RO (1998). Rumenic acid: a proposed common name for the major conjugated linoleic acid isomer found in natural products. Lipids.

[10] Lee KN, Kritchevsky D, Pariza MW (1994). Conjugated linoleic acid and atherosclerosis in rabbits. Atherosclerosis.

[11] Rahbar AR, Ostovar A, Derakhshandeh-Rishehri SM, Janani L, Rahbar A (2017). Effect of conjugated linoleic acid as a supplement or enrichment in foods on blood glucose and waist circumference in humans: A meta-analysis. Endocrine, Metabolic & Immune Disorders Drug Targets.

[12] Evans ME, Brown JM, McIntosh MK (2002). Isomer-specific effects of conjugated linoleic acid (CLA) on adiposity and lipid metabolism. J Nutr Biochem.

[13] Bawa S (2003). An update on the beneficial role of conjugated linoleic acid (CLA) in modulating human health: mechanisms of action – a review. Pol J Food Nutr Sci.

[14] Smedman A, Bengt V (2001). Conjugated linoleic acid supplementation in humans - metabolic effects. Lipids.

[15] Jiang J, Wolk A, Vessby B (1999). Relation between the intake of milk fat and the occurrence of conjugated linoleic acid in human adipose tissue. Am J Clin Nutr.

[16] Herbel BK, McGuire MK, McGuire MA, Shultz TD (1998). Safflower oil consumption does not increase plasma conjugated linoleic acid concentration in humans. Am J Clin Nutr.

[17] Adlof RO, Duwal S, Emken EA (2000). Biosynthesis of conjugated linoleic acid in humans. Lipids.

[18] Turpeinen AM, Mutanen M, Aro A, Salminen I, Basu S, Palmquist DL, Griinari JM (2002). Bioconversion of vaccenic acid to conjugated linoleic acid in human. Am J Clin Nutr.

[19] Moutsioulis AA, Rule DC, Murrieta CM, Bauman DE, Lock AL, Barbaro DM, Carey GB (2008). Human breast milk enrichment in conjugated linoleic acid after consumption of a conjugated linoleic acid–rich food product: a pilot study. Nutr Res.

[20] PN-EN ISO 1211:2011 Milk - Determination of Fat Content - Gravimetric Method. (In Polish).

[21] EN:ISO 5509:2000 Animal and vegetable fats and oils Preparation of methyl esters of fatty acids.

[22] Smedman AE, Gustafsson IB, Berglund LG, Vessby BO (1999). Pentadecanoic acid in serum as a marker for intake of milk fat: relations between intake of milk fat and metabolic risk factors. Am J Clin Nutr.

[23] Wu Z, Palmquist L (1991). Synthesis and biohydrogenation of fatty acids by ruminal microorganisms in vitro. J Dairy Scie.

[24] Masters N, McGuire MA, Beerman KA, Dasgupta N (2002). Maternal supplementation with CLA decreases milk fat in humans Lipids.

[25] Larsen TM, Toubro S, Astrup A (2003). Efficacy and safety of dietary supplements containing CLA for the treatment of obesity evidence from animal and human studies. J Lipid Res.

[26] Wahle KWJ, Heys SD, Rotondo D (2004). Conjugated linoleic acids: are they beneficial or detrimental to health?. Progress in Lipid Res.

[27] Mojska H, Socha P, Socha J, Soplińska E (2003). Trans fatty acids in human milk in Poland and their association with breastfeeding mothers’ diets. Acta Paediatrica.

